# Polarization of the epithelial layer and apical localization of integrins are required for engulfment of apoptotic cells in the *Drosophila* ovary

**DOI:** 10.1242/dmm.021998

**Published:** 2015-12-01

**Authors:** Tracy L. Meehan, Sarah E. Kleinsorge, Allison K. Timmons, Jeffrey D. Taylor, Kimberly McCall

**Affiliations:** Department of Biology, Boston University, 5 Cummington Mall, Boston, MA 02215, USA

**Keywords:** *Drosophila*, Integrin, Phagocytosis, Epithelial cell, Apoptosis, Ovary, Polarity, Migration

## Abstract

Inefficient clearance of dead cells or debris by epithelial cells can lead to or exacerbate debilitating conditions such as retinitis pigmentosa, macular degeneration, chronic obstructive pulmonary disease and asthma. Despite the importance of engulfment by epithelial cells, little is known about the molecular changes that are required within these cells. The misregulation of integrins has previously been associated with disease states, suggesting that a better understanding of the regulation of receptor trafficking could be key to treating diseases caused by defects in phagocytosis. Here, we demonstrate that the integrin heterodimer αPS3/βPS becomes apically enriched and is required for engulfment by the epithelial follicle cells of the *Drosophila* ovary. We found that integrin heterodimer localization and function is largely directed by the α-subunit. Moreover, proper cell polarity promotes asymmetric integrin enrichment, suggesting that αPS3/βPS trafficking occurs in a polarized fashion. We show that several genes previously known for their roles in trafficking and cell migration are also required for engulfment. Moreover, as in mammals, the same α-integrin subunit is required by professional and non-professional phagocytes and migrating cells in *Drosophila*. Our findings suggest that migrating and engulfing cells use common machinery, and demonstrate a crucial role for integrin function and polarized trafficking of integrin subunits during engulfment. This study also establishes the epithelial follicle cells of the *Drosophila* ovary as a powerful model for understanding the molecular changes required for engulfment by a polarized epithelium.

## Abstract (precis)

**Summary:** Apical integrin localization, mediated by polarized and directed trafficking, is crucial for proper engulfment by epithelial cells.

## INTRODUCTION

Epithelial cells, such as the retinal pigment epithelium (RPE) and bronchial epithelial cells, are required for engulfment on a daily basis. Engulfment in an epithelial layer requires substantial changes, all of which must be tightly regulated. However, very little is known about the molecular changes required for an epithelial layer to initiate engulfment. Integrins are αβ heterodimeric receptors that are crucial for phagocytosis in both professional and non-professional phagocytes in mammals (Finnemann and Silverstein, 2001; Sexton et al., 2001; Hsieh et al., 2012; Flannagan et al., 2014). Mammals have 18 α- and 8 β-subunits, but only use one specific α-subunit, αν, for engulfment by professional and non-professional phagocytes. Interestingly, in *Caenorhabditis elegans*, two different α-subunits are used for engulfment (Hsu and Wu, 2010; Hsieh et al., 2012). In *Drosophila*, there are five α- and two β-subunits. Recently, the integrin alpha-PS3 (αPS3)/βν heterodimer was shown to be required in professional phagocytes for the engulfment of bacteria and apoptotic cells (Shiratsuchi et al., 2012; Nonaka et al., 2013), but a role for integrins in non-professional phagocytes in *Drosophila* has not yet been demonstrated.

One example of an epithelium that is required for engulfment is the mammalian retinal pigment epithelium (RPE), cells of which engulf photoreceptor outer segments via their apical surface (Kevany and Palczewski, 2010). ανβ5, the integrin heterodimer associated with phagocytosis, is apically localized in RPE cells (Finnemann et al., 1997; Nandrot et al., 2008), despite basal localization of integrins in most cells. This suggests that heterodimer localization might be important for function. However, it is currently unknown how integrin heterodimers are asymmetrically localized within an engulfing cell.

One possibility is that cellular polarity is important for integrin trafficking, as it often is in migrating cells. The RPE cells are highly polarized throughout development and engulfment (Marmorstein, 2001), suggesting that this might be the case. Interestingly, the epithelial follicle cells (FCs) in the *Drosophila* ovary are also highly polarized (Tanentzapf et al., 2000; Morais-de-Sa et al., 2010; Fletcher et al., 2012) and engulf apoptotic debris via their apical side (Giorgi and Deri, 1976; Mazzalupo and Cooley, 2006; Tanner et al., 2011; Etchegaray et al., 2012). A second possibility is that integrin heterodimers are trafficked in a directed fashion. We tested both of these possibilities in this study and found that both play a role in engulfment and integrin trafficking.

The *Drosophila* ovary serves as a model of inducible engulfment by epithelial cells, which can provide insight into how a phagocytic state is activated in non-professional phagocytes (Thomson et al., 2010; Thomson and Johnson, 2010; Tanner et al., 2011; Etchegaray et al., 2012; Pritchett and McCall, 2012). Here, we demonstrate that integrins are required for engulfment by FCs and we show how they are apically trafficked in an adherent, epithelial cell layer. We found that integrin heterodimer localization and function is largely directed by the α-subunit, and apical localization of the αPS3/integrin beta-PS (βPS) heterodimer is required for engulfment. We found that many of the genes required for integrin trafficking in migratory cells are also required during engulfment, suggesting that migrating and engulfing cells might share common machinery. Moreover, our findings suggest that the *Drosophila* ovary might serve as an excellent *in vivo* model for integrin trafficking and function within the polarized RPE cells: our results indicate a high degree of similarity between these two tissues. Thus, the information gained here about the molecular changes within an engulfing epithelium might provide valuable insight into treatment for diseases such as macular degeneration and retinitis pigmentosa.

**TRANSLATIONAL IMPACT**

**Clinical issue**

Epithelial cells, such as retinal pigment epithelium cells and bronchial epithelial cells, continuously clear dead cells and debris from tissues. Failure of this engulfment process can lead to or exacerbate debilitating conditions such as retinitis pigmentosa, macular degeneration and asthma. However, little is known about the molecular changes that are required for an epithelial layer to initiate engulfment. To date, much of the research undertaken to understand engulfment has focused on identifying the genes required for engulfment. For example, it is well-established that integrins are αβ heterodimeric receptors that are required for phagocytosis in both professional and non-professional phagocytes in mammals. However, the pathways required for the regulation of engulfment receptors *in vivo* remain poorly understood.

**Results**

In the *Drosophila* ovary, germline debris produced by starvation-induced cell death at specific stages during oogenesis is engulfed by adjacent epithelial follicle cells. Here, the authors use this model of inducible engulfment by epithelial cells and the powerful genetic tools available in *Drosophila* to study the regulation of engulfment receptors *in vivo*. They show that integrins are required specifically on the apical surface of the epithelial follicle cells of the *Drosophila* ovary for engulfment. Through the use of RNA interference (RNAi) screens, they identify a requirement for polarization in engulfment and integrin trafficking, and show that genes involved in directed trafficking of integrins are also required for engulfment. Notably, many of these genes have no previously reported role in engulfment, but have been shown to have a role in migration.

**Implications and future directions**

Together, these results identify several genes required for integrin trafficking and regulation in an engulfing epithelial layer and suggest that engulfing and migrating cells share common trafficking machinery. These findings also establish the *Drosophila* ovary as a powerful model that shares many characteristics with mammalian retinal pigment epithelium cells for studying the molecular changes within an engulfing epithelium. Interestingly, genes involved in vesicle transport have been identified as candidate disease genes in retinitis pigmentosa, and the pathology of this condition is often attributed to the mislocalization of rhodopsin within photoreceptor cells. However, these findings suggest that some of the transport genes implicated in retinitis pigmentosa might instead be required for receptor trafficking within the engulfing epithelial layer. Further studies on the regulation of engulfment in the *Drosophila* ovary should improve our understanding of diseases in which engulfment by epithelial cells is defective.

## RESULTS

### The integrin subunits αPS3 and βPS become apically enriched in engulfing FCs

**Fig. 1.**
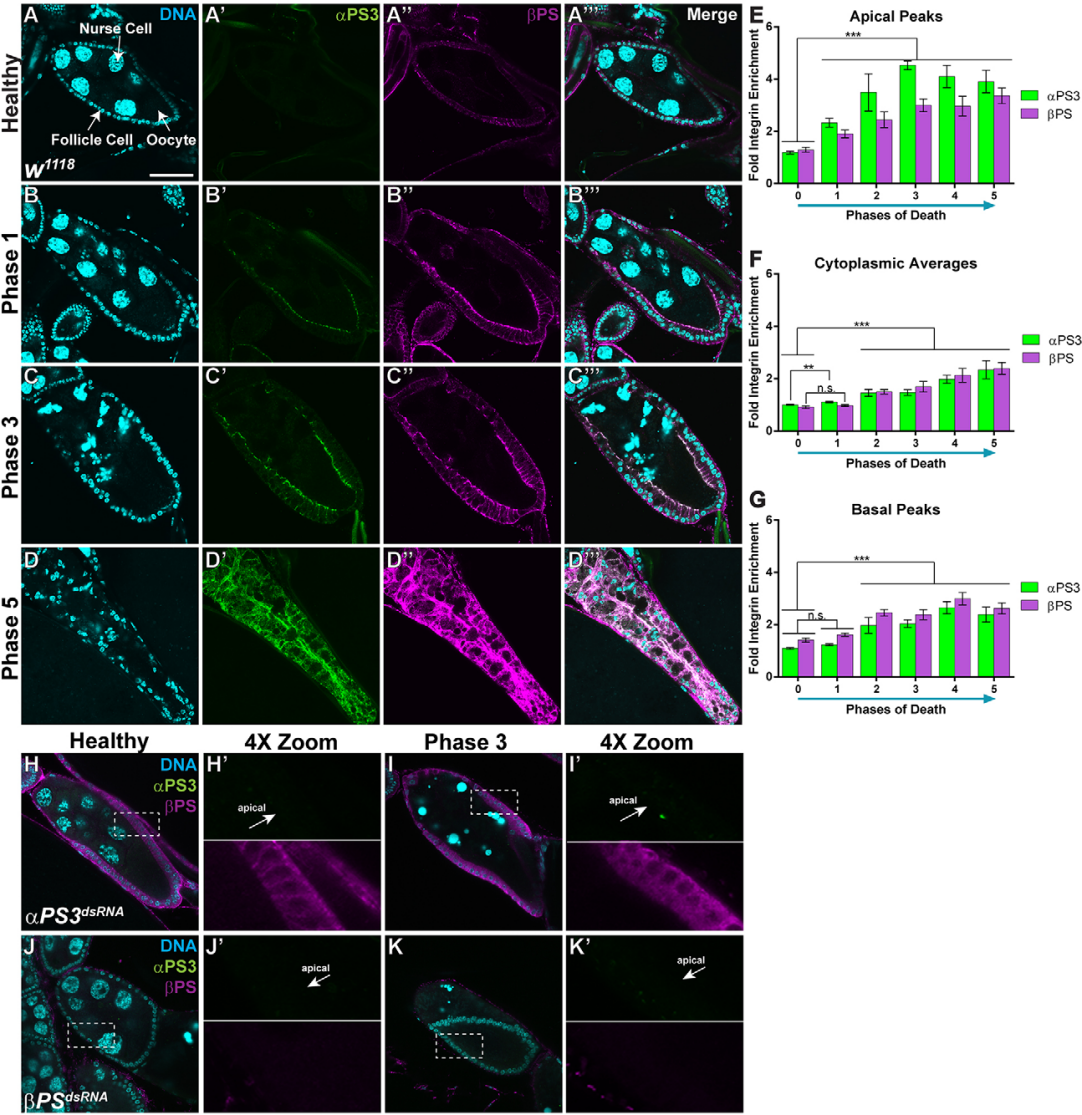
**The integrin heterodimer pair αPS3/βPS is apically enriched on follicle cells during engulfment.** (A-D) Healthy and progressively dying wild-type egg chambers from starved flies are labeled with DAPI (cyan), and antibodies against αPS3 (green) and βPS (magenta). Throughout the paper, healthy (phase 0), and phase 1, 3 and 5 egg chambers are shown. (A) Healthy (phase 0) egg chambers contain 15 germline-derived nurse cells, an oocyte, and are surrounded by an epithelial monolayer of follicle cells (FCs). (B) Phase 1 egg chambers have minor chromatin changes within the nurse cells. (C) Phase 3 egg chambers are characterized by condensed balls of nurse cell chromatin. (D) Phase 5 egg chambers contain few nurse cell nuclear fragments. (A) In healthy egg chambers, there is little to no αPS3 (A′) but low levels of βPS (A″) on all surfaces of the FCs. (B) In phase 1 egg chambers, αPS3 (B′) and βPS (B″) are enriched specifically on the apical surface of the FCs. (C) In phase 3 egg chambers, αPS3 (C′) and βPS (C″) increase more on the apical surface, and start increasing on the lateral and basal surfaces as well. (D) In phase 5 egg chambers, αPS3 (D′) and βPS (D″) continue to increase on all surfaces of the FCs. (E-G) Quantification of αPS3 and βPS in the apical and basal regions, and within the cytoplasm (see Materials and Methods). (E) The amount of αPS3 and βPS in the apical region increases significantly in phase 1 egg chambers and continues to increase throughout engulfment. (F) The amount of αPS3 and βPS in the cytoplasm starts to noticeably increase in phase 2 and continues increasing throughout engulfment. (G) The amount of αPS3 and βPS in the basal region also starts to noticeably increase in phase 2 and continues increasing throughout engulfment. (H-I′) Egg chambers expressing *αPS3^dsRNA^* have no detectable αPS3 and (I-I′) βPS does not become apically enriched during engulfment. Zooms in H′-K′ show αPS3 on the top and βPS on the bottom. (J-K′) Egg chambers expressing *βPS^dsRNA^* have no detectable αPS3 or βPS in healthy or dying egg chambers. All data are mean±s.e.m. For E-G, at least four egg chambers were quantified for each phase and a total of 63 were quantified over the five phases. One-way ANOVA and Bonferroni-Holm post-hoc tests were performed: ***P<0.005, **P<0.01. Scale bar: 50 µm.

Starvation-induced cell death occurs specifically during stages 7-9 of oogenesis (Giorgi and Deri, 1976), and five phases of death have been defined based on the progressive condensation and fragmentation of apoptotic germline-derived nurse cell nuclei (Etchegaray et al., 2012) (Fig. 1A-D). In healthy egg chambers, nurse cell chromatin is dispersed (Fig. 1A), but becomes disorganized in very early dying, or phase 1, egg chambers (Fig. 1B). By phase 3 of death, the nurse cell chromatin has become highly condensed in balls (Fig. 1C) and, by phase 5 of death, most nurse cell chromatin has been removed (Fig. 1D). Few hemocytes circulate within the ovary (King, 1970) and engulfment of germline debris is carried out by adjacent epithelial FCs (Giorgi and Deri, 1976).

We previously showed that the engulfment receptor Draper and the JNK pathway are required for engulfment by FCs (Etchegaray et al., 2012). In other systems, Draper and its orthologs work in parallel to other engulfment genes, including integrins (Nagaosa et al., 2011; Hsieh et al., 2012; Kim et al., 2012; Shiratsuchi et al., 2012). To investigate whether integrins participate during engulfment in the ovary, we analyzed the expression of integrin subunits during engulfment utilizing previously made antibodies (Brower et al., 1984; Wada et al., 2007; Nagaosa et al., 2011). It has previously been shown that αPS1, αPS2 and βPS are expressed throughout oogenesis, whereas αPS3, αPS4 and αPS5 become transcriptionally upregulated during late oogenesis (Dinkins et al., 2008). We found that the expression pattern and intensity of αPS1, αPS2 and βν did not change during engulfment (data not shown), but αPS3 and βPS increased strikingly. In healthy mid-stage (stages 7-9) egg chambers, αPS3 was not detected on the FCs (Fig. 1A′) and βPS was present on all surfaces (Fig. 1A″) (Dinkins et al., 2008). Both subunits increased specifically on the apical surface of FCs in very early dying, phase 1, egg chambers (Fig. 1B′,B″). We quantified the intensity of αPS3 and βPS throughout the cell (Fig. S1A-F and Fig. 1E-G), and found that the intensity of αPS3 increased more than twofold on the apical surface (Fig. 1E) of phase 1 egg chambers, whereas there was only a 1.2-fold increase on the basal surface (Fig. 1G). Both αPS3 and βPS continued to increase on the apical surface as engulfment proceeded (phases 3-5), but also started to increase on the basal surface and within the cytoplasm to a lesser extent (Fig. 1C′,C″,D′,D″,E-G). The colocalization of these two subunits suggests that αPS3 and βPS form a heterodimer on the apical surface during engulfment.

### The integrin heterodimer αPS3/βPS is required specifically in FCs during engulfment

To determine whether αPS3 and βPS were required in the FCs for engulfment, we knocked down αPS3 and βPS using RNA interference (RNAi) and confirmed that there was effective knockdown using antibody staining (Fig. 1H-K). We expressed dsRNA against integrin subunits using a *GAL4* driver (*GR1-GAL4*) that begins to be expressed in mid-oogenesis (Etchegaray et al., 2012), allowing for integrin expression and establishment of a normal follicular epithelium in early oogenesis. When well-fed, loss of αPS3 and βPS in FCs did not disrupt normal development of the egg chambers. Loss of βPS resulted in round late-stage egg chambers with short dorsal appendages, as previously shown (Bateman et al., 2001), but the chorion formed normally, indicating that FCs functioned normally under these conditions.

**Fig. 2.**
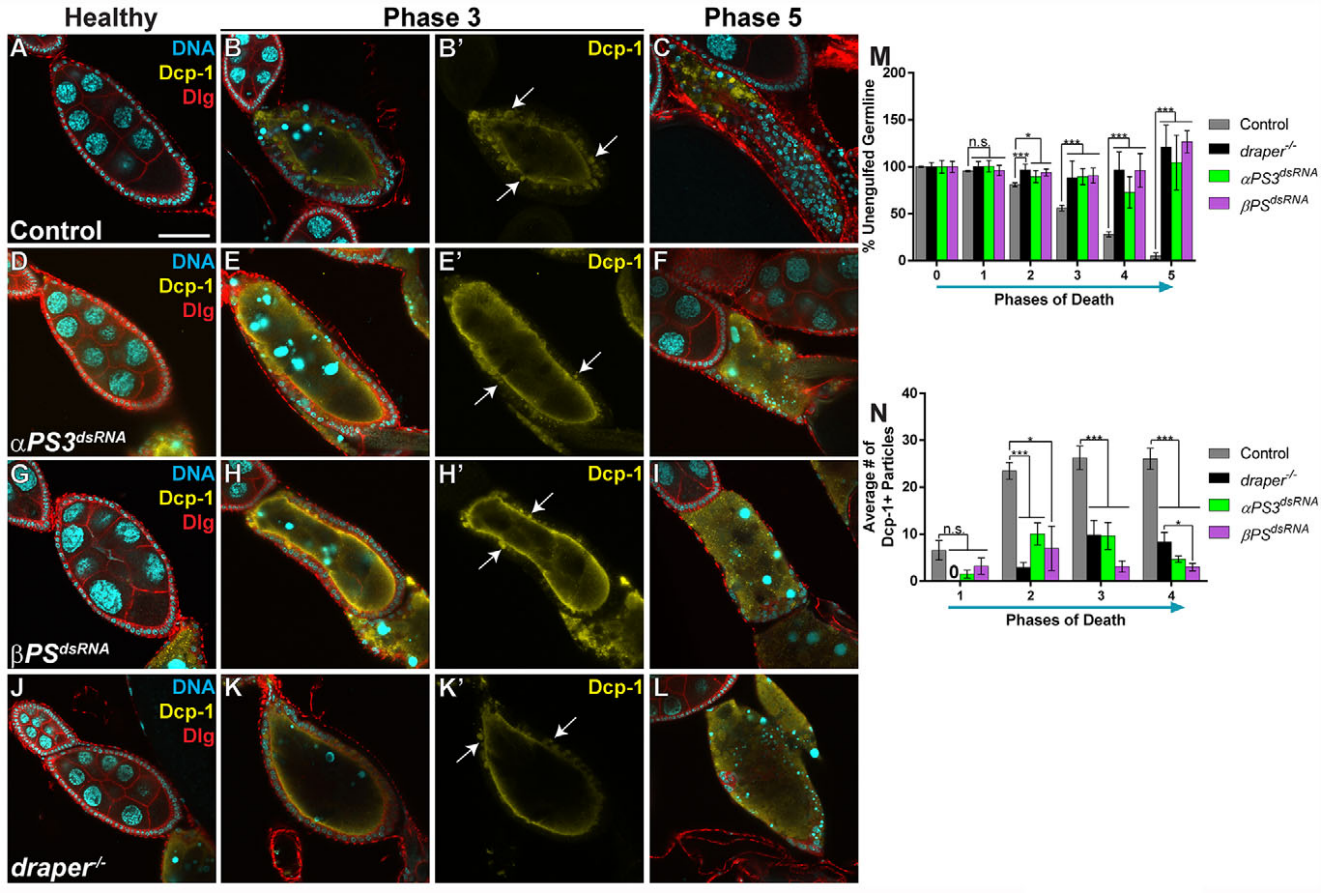
**The integrin heterodimer pair αPS3/βPS is required in follicle cells for engulfment.** (A-L) Egg chambers from starved flies are labeled with DAPI (cyan), cleaved anti-Dcp-1 (yellow) and anti-Dlg (red) to visualize membranes. Antibody against cleaved Dcp-1 specifically marks the dying germline and engulfed material. (A-C) Control (*GR1-GAL4, G89/TM6B*) egg chambers show normal death and engulfment. (A) Healthy egg chambers do not label with Dcp-1. (B,B′) A phase 3 dying egg chamber shows several Dcp-1-positive particles (arrows) inside the follicle cells (FCs), indicating engulfment. (C) A phase 5 dying egg chamber shows little remaining germline, and few Dcp-1-positive particles still inside the FCs. (D-F) Egg chambers expressing *αPS3^dsRNA^* specifically in the FCs are normal when healthy (D) but have little to no particle uptake in phase 3 (E,E′; arrows). (F) Loss of *αPS3* in the FCs results in lingering germline material by phase 5. (G-I) Egg chambers expressing *βPS^dsRNA^* specifically in the FCs are similar to those with loss of *αPS3*, with few particles (H,H′; arrows) and lingering germline material (I). (J-L) *draper^−/−^* egg chambers (Etchegaray et al., 2012) are normal when healthy (J) but have little to no particle uptake in phase 3 (K,K′; arrows). (L) Loss of *draper* in the FCs results in lingering germline material. (M) Quantification of unengulfed germline. (N) Quantification of the number of Dcp-1-positive particles in phases 1-4. All data are mean±s.e.m. At least three egg chambers were quantified for each genotype, phase and quantification method in M,N. For M, 238 egg chambers were quantified for the control (a mixture of *GR1-GAL4, G89* and sibling controls); 57 for *αPS3^dsRNA^*; 110 for *βPS^dsRNA^*; and 121 for *draper^−/−^*. For N, 103 egg chambers were quantified for the control, 33 for *αPS3^dsRNA^*, 33 for *βPS^dsRNA^* and 44 for *draper^−/−^*. One-way ANOVA and Bonferroni-Holm post-hoc tests were performed: ***P<0.005, *P<0.05, n.s., non-significant. Scale bar: 50 µm.

To visualize engulfment, flies expressing *αPS3* and *βPS* dsRNA under the control of *GR1-GAL4* were starved and ovaries were stained with cellular markers (Fig. 2) (Etchegaray et al., 2012; Sarkissian et al., 2014). Whereas healthy egg chambers were normal with RNAi knockdown (Fig. 2A,D,G), loss of either subunit resulted in defective FC enlargement and engulfment in dying egg chambers compared to controls (Fig. 2B-C,E-F,H-I). Specifically, FCs failed to enlarge and engulf during phase 3, and FCs disappeared by phase 5, leaving behind unengulfed germline cytoplasm and highly condensed nurse cell chromatin, resembling *draper* mutants (Fig. 2K-L). As previously described (Etchegaray et al., 2012), we quantified the percentage of unengulfed germline using membrane markers to measure the area of the germline and the total egg chamber (Fig. 2M), and found that integrin knockdowns had engulfment defects similar to *draper* mutants. As another measurement of engulfment, we used an antibody raised against cleaved caspase Dcp-1, which marks the germline of dying egg chambers and engulfed apoptotic debris (Sarkissian et al., 2014) (Fig. 2A-L). We used this antibody to specifically count the discrete number of engulfed particles within the FCs. Healthy egg chambers did not label for cleaved Dcp-1 (Fig. 2A,D,G,J), but control dying egg chambers showed particle uptake beginning in phase 1, which increased and plateaued by phase 3 (Fig. 2N). However, the loss of *draper*, *αPS3* or *βPS* resulted in defective particle uptake when compared to controls (Fig. 2N). This defect is most clearly seen in the images showing only the Dcp-1 channel (Fig. 2B′,E′,H′,K′). By phase 4, *draper* mutants contained more particles than the integrin knockdowns (Fig. 2N), suggesting that both *draper* and integrins are required for uptake but *draper* might also have defects in phagosome maturation, whereas integrins do not. Indeed, Draper and its ortholog in *C. elegans*, Ced-1, have been shown in multiple cell types to play a role in cell corpse processing (Kurant et al., 2008; Yu et al., 2008; Lu et al., 2011; Chen et al., 2013; Han et al., 2014; Evans et al., 2015). We also found that *draper* was not required for enrichment of αPS3/βPS (Fig. S2A-D′), and the integrin pathway was not required for enrichment of Draper or activation of the JNK pathway (Fig. S2E-L), indicating that enrichment of integrins and Draper, and activation of the JNK pathway, occur independently of each other.

### The localization and function of αPS3/βPS is largely directed by αPS3

**Fig. 3.**
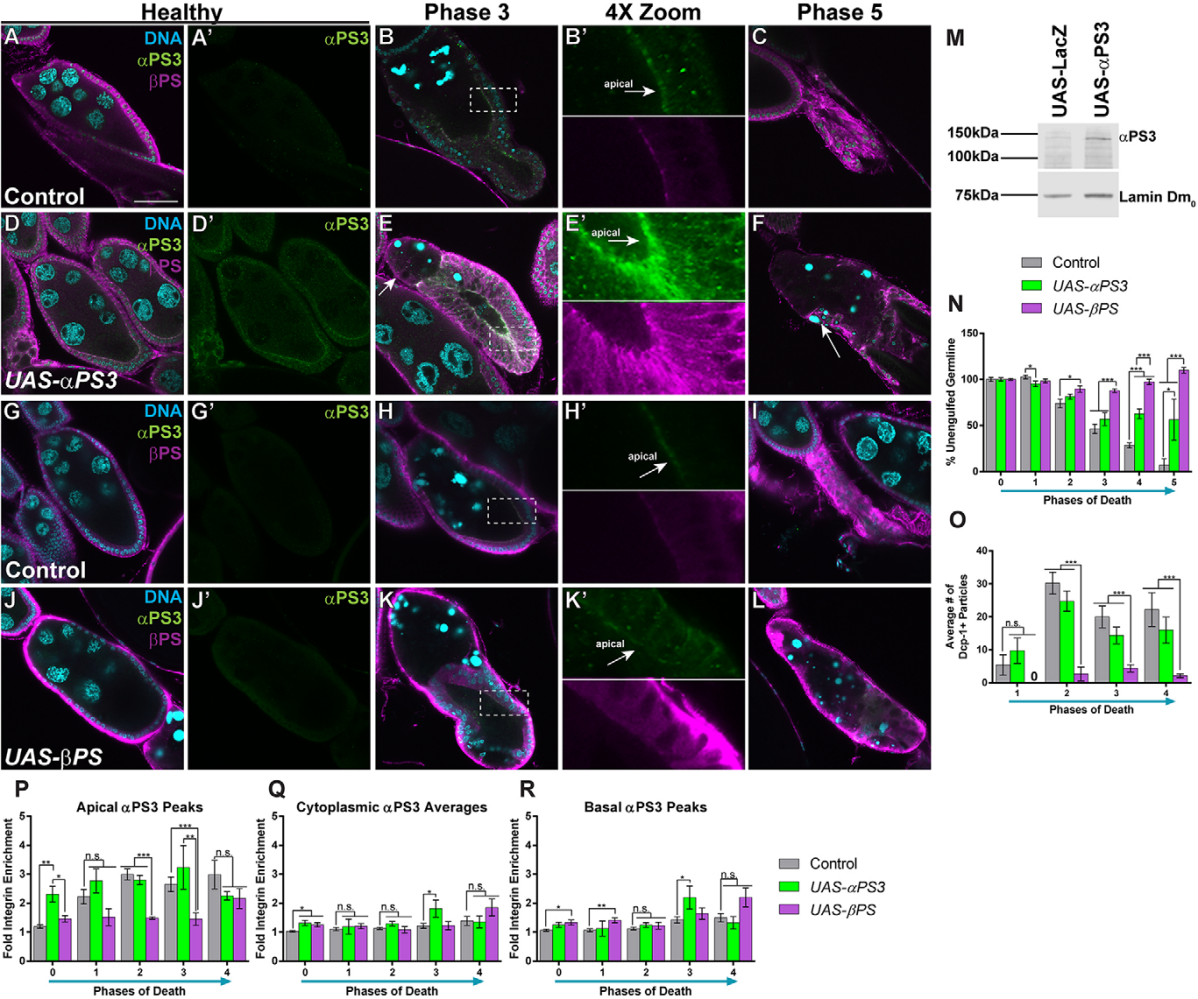
**The α-subunit directs the localization and function of the heterodimer.** (A-L) Egg chambers from starved flies stained with DAPI (cyan), anti-αPS3 (green) and anti-βPS (magenta). Boxed regions in the phase 3 images are shown in the 4× zoom. Arrows in 4× zooms indicate the apical surface of the follicle cells. (A-C) Sibling control (*UAS-αPS3/+; +/TM6B)* egg chambers show normal cell death, engulfment and integrin enrichment. (D-F) Egg chambers expressing *UAS-αPS3* show largely normal engulfment. (D) Healthy egg chambers have premature αPS3 (D′) and βPS on the apical surface. (E,E′) Phase 3 dying egg chambers have αPS3 and βPS on the apical surface and inappropriate migration of anterior follicle cells (FCs; arrow in E). (F) *UAS-αPS3* phase 5 egg chambers successfully engulf the posterior germline (arrow). (G-I) Sibling control (*UAS- βPS/+; +/TM6B*) egg chambers show normal cell death, engulfment and integrin enrichment. (J-L) Egg chambers expressing *UAS-βPS* are engulfment-defective. (J) Healthy egg chambers show enrichment of βPS on the basal surface of the FCs and no premature expression of αPS3 (J′). (K,K′) Phase 3 egg chambers show minimal enlargement and engulfment. (L) *UAS-βPS* results in engulfment-defective egg chambers. (M) A western blot of conditioned whole *UAS-αPS3* ovaries shows a robust increase in αPS3 when compared to a *UAS-lacZ* control. (N) Quantification of unengulfed germline. (O) Quantification of the number of Dcp-1-positive particles in phases 1-4. (P-R) Quantification of αPS3 and βPS in the apical and basal regions, and within the cytoplasm. All data are mean±s.e.m. At least three egg chambers were quantified for each genotype, phase and quantification method in N-R. For N, 42 egg chambers were quantified for the control, 34 for *UAS-αPS3* and 131 for *UAS-βPS*. For O, 31 egg chambers were quantified for the control, 21 for *UAS-αPS3* and 49 for *UAS-βPS*. For P-R, 53 egg chambers were quantified for the control, 37 for *UAS-αPS3* and 43 for *UAS-βPS*. One-way ANOVA and Bonferroni-Holm post-hoc tests were performed: ***P<0.005, **P<0.01, *P<0.05, n.s., non-significant. Scale bar: 50 µm.

Given the polarized localization of integrins in our system, we manipulated expression levels of αPS3 and βPS to determine which subunit dictates the heterodimer localization. Overexpression of αPS3 in FCs led to a robust increase in levels of the protein in whole ovaries (Fig. 3M) and a premature twofold apical enrichment of αPS3/βPS in healthy egg chambers (Fig. 3A,A′,D,D′), equivalent to phase 1 control egg chamber enrichment (Fig. 3P). Overall, αPS3-expressing egg chambers engulfed normally (Fig. 3E,O), but often terminated with lingering germline material owing to premature FC migration (Fig. 3F,N). During oogenesis, some of the anterior FCs normally migrate toward the posterior, beginning in stage 9 (King, 1970). In many egg chambers overexpressing αPS3, all of the anterior FCs migrated to the posterior, leaving part of the egg chamber without FCs (Fig. 3E, arrow). Despite the lingering germline, the number of engulfed particles indicated that the egg chambers overexpressing αPS3 were not engulfment-defective (Fig. 3O).

Surprisingly, we found that egg chambers overexpressing βPS were engulfment-defective (Fig. 3K-L,N,O) and had considerable basal enrichment of βPS in healthy (Fig. 3J) and dying (Fig. 3K,K′) egg chambers. Throughout engulfment, overexpression of βPS prevented apical enrichment of αPS3 until phase 4 (Fig. 3P), suggesting that overexpression of βPS inhibits αPS3/βPS apical trafficking. Together, these results suggest that the αPS3 subunit determines the apical localization and function of the heterodimer, and basal localization of βPS has an inhibitory effect on engulfment. Consistent with this, loss of αPS3 was associated with normal levels of βPS within healthy egg chambers (Fig. 1H), but βPS did not become apically enriched in dying egg chambers (Fig. 1I,I′). In addition, *βPS^dsRNA^*-expressing egg chambers had no detectable αPS3 or βPS in the FCs (Fig. 1J-K′), suggesting that αPS3 expression is dependent on βPS. Because of the importance of αPS3 in directing the localization and function of the heterodimer, we screened candidate genes that might regulate αPS3 localization.

### The maintenance of a polarized epithelium is required for engulfment

**Fig. 4.**
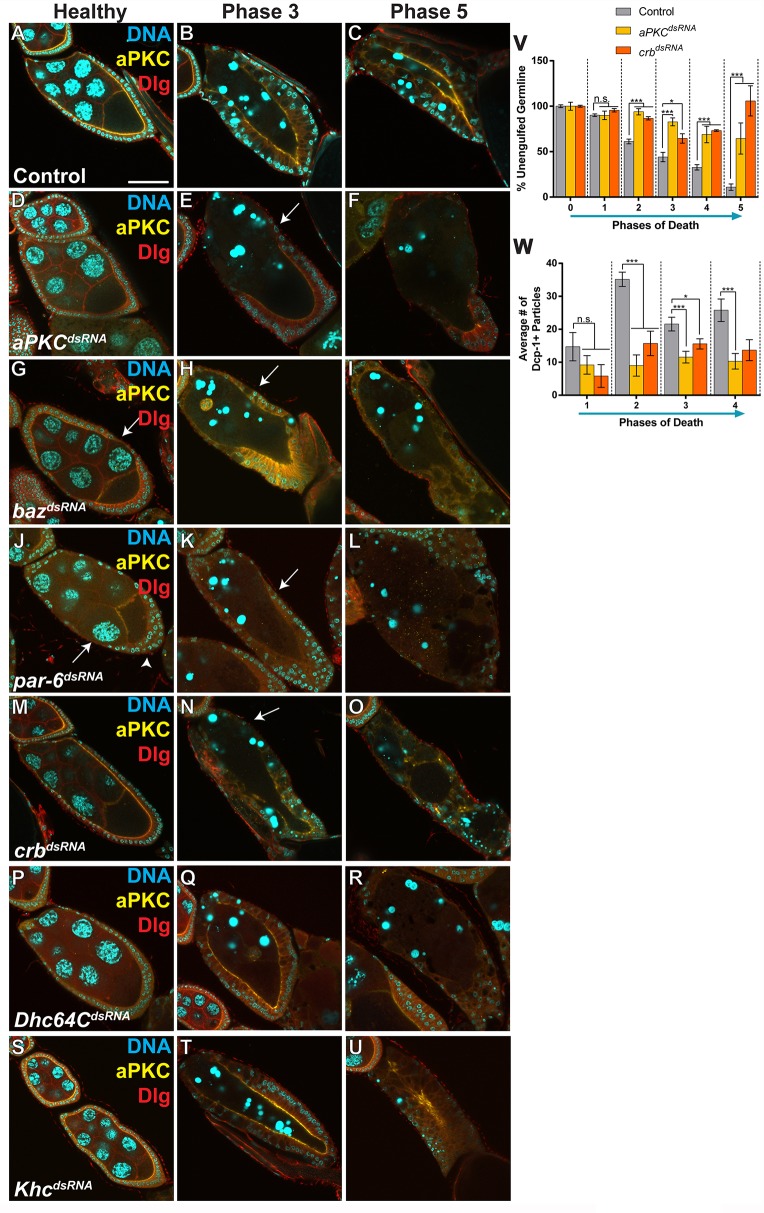
**Apical-basal polarity is required for engulfment.** (A-U) Egg chambers from starved flies stained with DAPI (cyan), anti-aPKC (yellow) and anti-Dlg (red). (A-C) Healthy, phase 3 and phase 5 control (*tub-Gal80/+; GR1-GAL4/UAS-eGFP^dsRNA^*) egg chambers show follicle cell (FC) enlargement and maintain apical localization of aPKC as engulfment progresses. (D-F) *aPKC^dsRNA^* healthy, phase 3 and phase 5 egg chambers show a reduced amount of aPKC localization, minimal FC enlargement, and defective engulfment. (G-I) *baz^dsRNA^* healthy and phase 5 egg chambers show a reduced amount of aPKC localization, and defective engulfment, whereas phase 3 egg chambers show increased, but mislocalized, aPKC. (J-L) *par-6^dsRNA^* healthy, phase 3 and phase 5 egg chambers show a loss in aPKC localization, no FC enlargement, and defective engulfment. Premature migration of FCs to the posterior of the egg chamber was seen in some healthy *par-6* egg chambers (J; arrowhead). Arrows in G and J indicate gaps in the follicle cell layer. (M-O) *crb^dsRNA^* healthy, phase 3 and phase 5 egg chambers show normal aPKC localization initially but, as engulfment progresses, aPKC localization is lost and the egg chambers are engulfment-defective. Arrows in E, H, K, N show regions without FC enlargement. (P-R) Egg chambers expressing *Dhc^dsRNA^* show aPKC localization in phase 3 egg chambers; however, the localization is lost by phase 5 and egg chambers are engulfment-defective. (S-U) Egg chambers expressing *Khc^dsRNA^* show normal aPKC localization and do not have engulfment defects. (V) Quantification of unengulfed germline for control, *aPKC^dsRNA^* and *crb^dsRNA^* egg chambers. (W) Quantification of the number of Dcp-1-positive particles in phases 1-4 for control, *aPKC^dsRNA^* and *crb^dsRNA^* egg chambers. At least three egg chambers were quantified for each genotype, phase and quantification method in V,W. 51 egg chambers were quantified for the control, 35 for *aPKC^dsRNA^* and 30 for *crb^dsRNA^*. All data are mean±s.e.m. One-way ANOVA and Bonferroni-Holm post-hoc tests were performed: ***P<0.005, *P<0.05, n.s., non-significant. Scale bar: 50 µm.

Given the apical enrichment of integrins during engulfment, we hypothesized that cell polarity would be essential for proper engulfment by the FCs. Atypical protein kinase C (aPKC), one of the factors that determines the apical domain of the FCs, was maintained throughout engulfment (Fig. 4A-C), suggesting that FC apical polarization is needed. To determine whether FC polarity was required for the engulfment of germline material, we performed FC-specific knockdowns of several apical factors that regulate cell polarity: *aPKC*, *baz*, *par-6* and *crb*. Again, we used *GR1-GAL4*, so that proper polarity would be established in the follicular epithelium in early oogenesis, and polarity would only be disrupted beginning in mid-oogenesis. When each knockdown line was well-fed, flies produced healthy stage-10 egg chambers with normal yolk deposition, showing that oogenesis was not grossly affected. However, when the lines were starved, some defects were seen. *aPKC^dsRNA^*-, *baz^dsRNA^*- and *par-6^dsRNA^*-expressing healthy egg chambers showed a reduced amount of aPKC and some egg chambers had a few gaps between FCs, but otherwise developed normally (Fig. 4D,G,J, arrows). A premature-migration defect was also observed in *par-6^dsRNA^-*expressing healthy egg chambers (starved or well-fed), where anterior FCs migrated towards the posterior early in stage-8 egg chambers, forming a double layer (Fig. 4J, arrowhead). *crb^dsRNA^-*expressing healthy egg chambers showed normal aPKC localization with no developmental defects (Fig. 4M).

In degenerating egg chambers, however, pronounced engulfment defects were observed in knockdowns of all of these apical factors (*aPKC*, *baz*, *par-6* and *crb*; Fig. 4E,F,H,I,K,L,N,O). Phase 3 dying egg chambers expressing *aPKC^dsRNA^*, *par-6^dsRNA^* or *crb^dsRNA^* showed reduced aPKC and defective FC enlargement (Fig. 4E,K,N, arrows). Phase 3 egg chambers expressing *baz^dsRNA^* were variable, but often showed increased but mislocalized aPKC (Fig. 4H), suggesting that Bazooka might inhibit aPKC as it does during apical constriction (David et al., 2013). In each knockdown line, the FCs that maintained any apical aPKC localization were able to enlarge. This demonstrates a need for aPKC for both the polarization of the FCs and the enlargement of the FCs during engulfment. All late dying (phase 5) egg chambers from each knockdown line showed a loss in aPKC localization, a loss of FCs, and large amounts of germline material remaining (Fig. 4F,I,L,O). This indicates that the knockdown of any one of the apical factors (*aPKC*, *baz*, *par-6* or *crb*) results in defective engulfment. Loss of *aPKC* and *crb* resulted in the strongest and most consistent engulfment-defective phenotype, so we analyzed these mutants further. Based on antibody staining, *aPKC^dsRNA^* produced a partial knockdown (Fig. 4A-F), but *crb^dsRNA^* produced a very strong knockdown (Fig. S3A-D). Both *aPKC^dsRNA^* and *crb^dsRNA^* showed strong defects in the percentage of unengulfed germline and the number of engulfed particles (Fig. 4V,W).

We also knocked down the two molecular motors that maintain cell polarity, Dynein and Kinesin. We verified the FC-specific knockdowns using antibody staining and found that both were strong, yet incomplete, knockdowns (Fig. S3E-L′). Absence of the Dynein heavy chain, *Dhc64C*, led to engulfment defects, but only after phase 3 (Fig. 4Q,R). *Dhc64C^dsRNA^-*expressing phase 5 egg chambers showed a loss in FCs, resulting in a large amount of lingering germline material. Interestingly, the Kinesin heavy chain (*Khc*) knockdown did not show engulfment defects and had phase 5 egg chambers with proper FC enlargement and clearance of germline material (Fig. 4T,U). Within the FCs, Dynein transports material along the microtubules towards the apical membrane and Kinesin transports material toward the basal membrane (Horne-Badovinac and Bilder, 2008). This suggests that apical molecular transport is essential for engulfment. Taken together, these findings show that apical–basal FC polarity establishment and maintenance is required for engulfment to occur properly in epithelial cells, identifying a previously undescribed role for cell polarity.

### Cell polarization is required for asymmetric integrin localization during engulfment

**Fig. 5.**
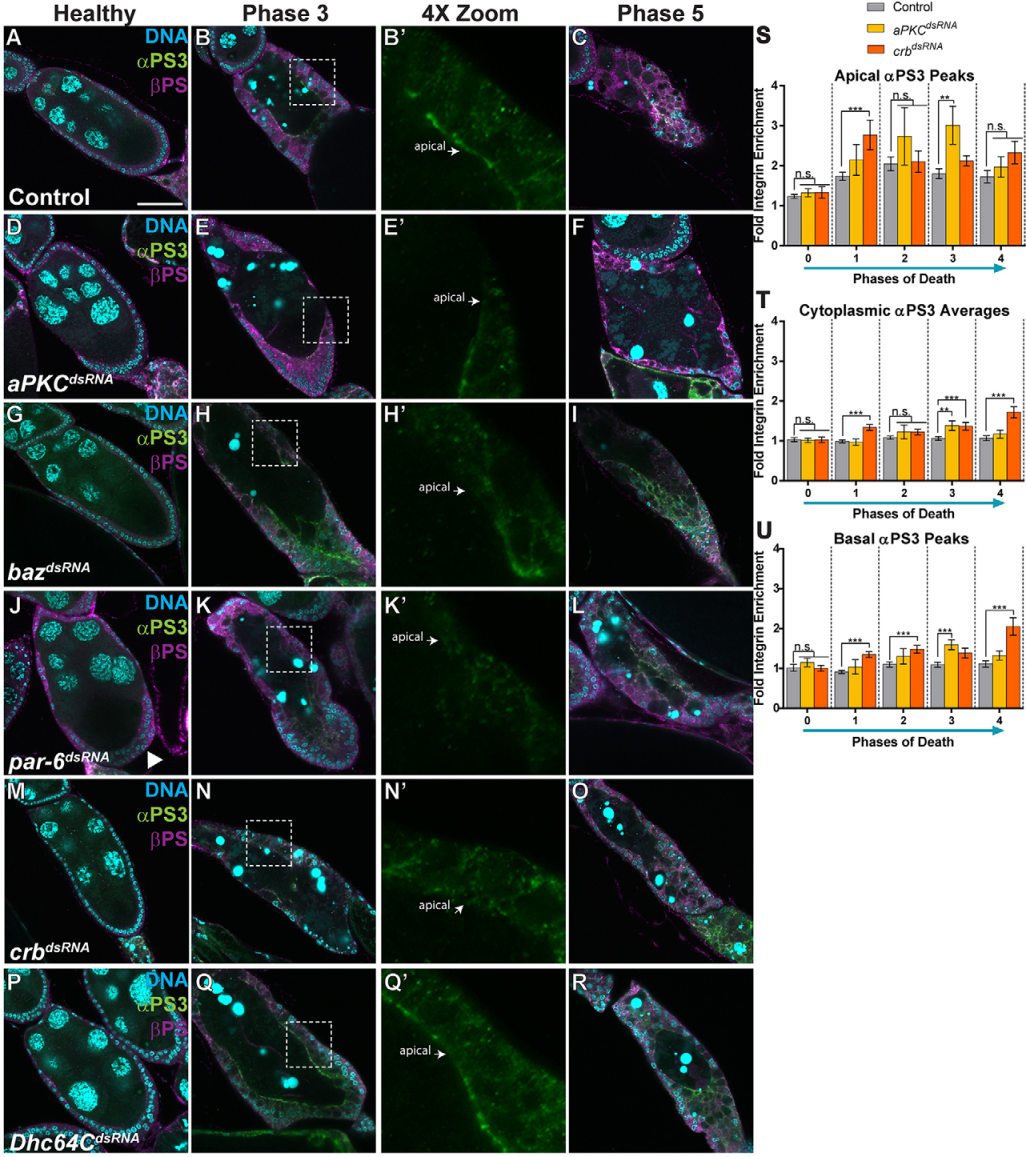
**Cell-polarity knockdown egg chambers are defective for integrin enrichment and engulfment.** (A-R) Egg chambers from starved flies stained with DAPI (cyan), anti-αPS3 (green) and anti-βPS (magenta). 4× zooms show the αPS3 channel of the boxed regions in the phase 3 images. Arrows in 4× zooms indicate the apical surface of the follicle cells. (A-C) Healthy, phase 3 and phase 5 control (*tub-Gal80/+; GR1-GAL4/UAS-eGFP^dsRNA^*) egg chambers show apical enrichment of αPS3/βPS as engulfment progresses. (D-O) Egg chambers expressing dsRNA against the apical determinants all show disrupted localization of αPS3. Arrowhead in J points to a double layer of FCs in a *par-6* healthy egg chamber. (P-R) Egg chambers expressing *Dhc64C^dsRNA^* show initial apical αPS3 enrichment (Q,Q′), which is later lost (R). (S-U) Quantification of αPS3 in the apical and basal regions, and within the cytoplasm, for control, *aPKC^dsRNA^* and *crb^dsRNA^* egg chambers. The high temperature (29°C) might inhibit normal αPS3/βPS enrichment somewhat compared to 25°C (Fig. 1). All data are mean±s.e.m. At least three egg chambers were quantified for each genotype and phase in S-U. 62 egg chambers were quantified for the control, 38 for *aPKC^dsRNA^* and 38 for *crb^dsRNA^*. One-way ANOVA and Bonferroni-Holm post-hoc tests were performed: ***P<0.005, **P<0.01, n.s., non-significant. Scale bar: 50 µm.

Because FC polarization was required for engulfment, we screened the cell-polarity knockdown lines to see whether they affected the apical enrichment of αPS3 and βPS compared to controls (Fig. 5A-C). We found that, when cell polarity genes were disrupted, βPS expression was normal in healthy egg chambers (Fig. 5D,G,J,M). Although αPS3/βPS increased during engulfment, neither subunit localized asymmetrically on the apical surface of the FCs (Fig. 5E,E′,H,H′,K,K′,N,N′). Instead, αPS3/βPS had a variable and uneven localization to multiple surfaces and throughout the cytoplasm (Fig. 5S-U). This variability was seen between egg chambers and between cells within individual egg chambers (Fig. S3M-R). When molecular transport was disrupted with the knockdown of Dhc64C, αPS3 and βPS were also mislocalized in the dying egg chambers (Fig. 5Q-R). This indicates that FC polarization promotes the asymmetric enrichment of the αPS3/βPS integrin heterodimer during engulfment. Without proper polarity, αPS3/βPS becomes mislocalized to all surfaces of the cell, suggesting that polarization sets up the directionality of integrin transport. Within these egg chambers, when αPS3 was basally localized, the FCs often did not enlarge or engulf, suggesting that basal localization of αPS3 can inhibit engulfment. This can be seen in egg chambers that had variable αPS3 localization in FCs that are side by side. Only the FCs with normal apical enrichment enlarged (Fig. 5, zooms). We also examined the localization of another known engulfment receptor, Draper, in the cell-polarity knockdown lines. We found that Draper enrichment was defective in FCs only in the aPKC knockdown line (Fig. S4C-D), perhaps owing to the importance of aPKC as a signaling component without the remainder of the apical complex (Baz and Par-6) (Kim et al., 2009). The other knockdown lines did not have noticeable defects in Draper localization (*baz^dsRNA^* shown as an example in Fig. S4E-F). This indicates that aPKC and cell-polarity genes might be necessary for the asymmetric localization of multiple engulfment receptors during engulfment.

### Migrating and engulfing cells utilize much of the same integrin trafficking machinery

**Fig. 6.**
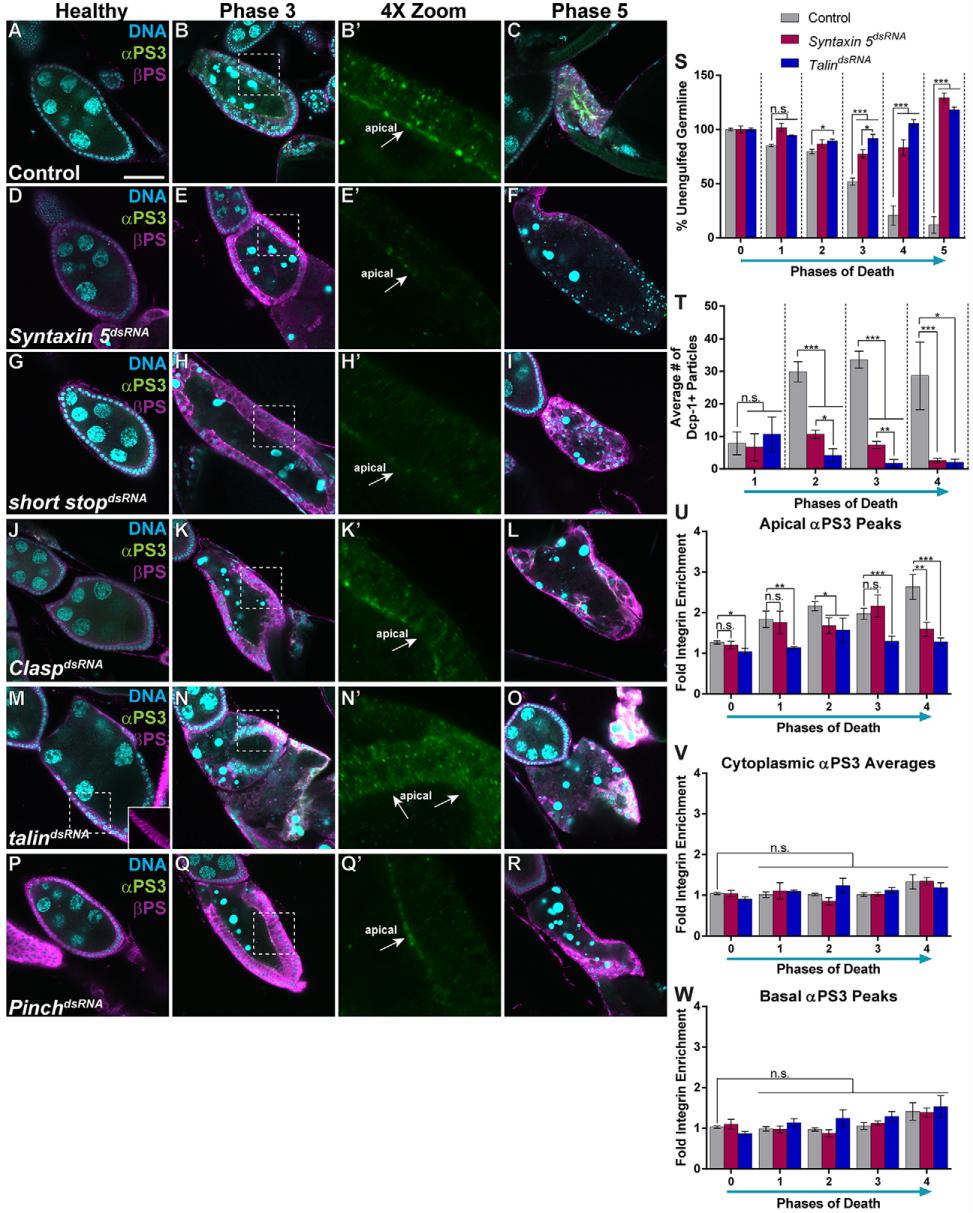
**Genes involved in trafficking from the Golgi and integrin signaling are required for integrin enrichment and engulfment.** (A-R) Egg chambers from starved flies stained with DAPI (cyan), anti-αPS3 (green) and anti-βPS (magenta). 4× zooms show the αPS3 channel of the boxed regions in the phase 3 images. (A-C) Control (*GR1-GAL4/UAS-luciferase^dsRNA^*) egg chambers show normal engulfment and integrin enrichment. (D-F) Egg chambers expressing *Syntaxin 5^dsRNA^* in the follicle cells (FCs) are engulfment-defective. (D) Healthy egg chambers have normal βPS localization. (E,E′) Phase 3 egg chambers show minimal apical enrichment of αPS3 and βPS. (F) Pyknotic nuclei suggest FC death in phase 5. (G-I) Egg chambers expressing *short stop^dsRNA^* in the FCs are engulfment-defective. (G) Healthy egg chambers show normal βPS localization. (H,H′) Phase 3 egg chambers show minimal apical enrichment of αPS3 and βPS. (J-L) Egg chambers expressing *Clasp^dsRN^*^A^ (also known as *chb*) in the FCs are engulfment-defective. (J) Healthy egg chambers show normal integrin expression and initially have normal enrichment of αPS3 to the apical surface. (K,K′) However, egg chambers expressing *Clasp^dsRNA^* did not enrich αPS3 on the apical surface as engulfment proceeded. (M-O) Egg chambers expressing *talin^dsRNA^* in the FCs are engulfment-defective. (M) Healthy egg chambers often have more basally localized βPS (inset). (N,N′) Phase 3 egg chambers show no αPS3 enrichment and mislocalization of βPS. (P-R) Egg chambers expressing *Pinch^dsRNA^* (also known as *stck*) in the FCs are engulfment-defective. (P) Healthy egg chambers have normal βPS localization. (Q,Q′) Phase 3 egg chambers have minimal apical enrichment but mislocalization of βPS. (S) Quantification of unengulfed germline. (T) Quantification of the number of Dcp-1-positive particles. (U-W) Quantification of αPS3 and βPS in the apical and basal regions, and within the cytoplasm. All data are mean±s.e.m. At least three egg chambers were quantified for each genotype, phase and quantification method in S-W. For S, 94 egg chambers were quantified for the control, 44 for *Syntaxin 5^dsRNA^* and 30 for *talin^dsRNA^*. For T, 55 egg chambers were quantified for the control, 21 for *Syntaxin 5^dsRNA^* and 16 for *talin^dsRNA^*. For U-W, 45 egg chambers were quantified for the control, 40 for *Syntaxin 5^dsRNA^* and 28 for *talin^dsRNA^*. One-way ANOVA and Bonferroni-Holm post-hoc tests were performed: ***P<0.005, **P<0.01, *P<0.05, n.s., non-significant. Scale bar: 50 µm.

Cell polarization is often required for integrin localization in migratory cells (Thapa and Anderson, 2012; Thapa et al., 2012). Interestingly, migration in border cells and during dorsal closure requires much of the engulfment machinery (Dinkins et al., 2008; Brock et al., 2012; Ellis et al., 2013), suggesting that common molecular processes are shared between migration and engulfment. We found it striking that the same α-subunit is used for both engulfment and migration in *Drosophila* and in mammals (Dinkins et al., 2008; Shiratsuchi et al., 2012; Nonaka et al., 2013; Law et al., 2015; Tsirmoula et al., 2015). Thus, given the link between cell polarity and integrins in migratory cells, and the fact that αPS3 is required in migratory and engulfing cells, we screened for engulfment and integrin-enrichment defects in knockdowns of several genes that are associated with migration and/or integrin signaling. We found that several genes associated with transport along microtubules and Golgi vesicle formation were required for engulfment and integrin enrichment: *Syntaxin 5*, *short stop* and *Clasp* (Fig. 6D-L). Loss of these genes did not disrupt βPS expression in healthy egg chambers (Fig. 6D,G,J) and had some initial enrichment of αPS3 during engulfment. As engulfment proceeded, however, αPS3 did not apically enrich to control levels (Fig. 6B,B′,E,E′,H,H′,K,K′). Loss of *Syntaxin 5*, *short stop* or *Clasp* resulted in engulfment-defective egg chambers (Fig. 6F,I,L).

Surprisingly, we also found that *talin* and *Pinch*, genes associated with integrin activation and downstream signaling (Honda et al., 2013), were required for integrin enrichment (Fig. 6M-R). Loss of *talin* resulted in increased basolateral βPS expression in healthy egg chambers (Fig. 6M, inset) and a lack of αPS3 enrichment on any surface in dying egg chambers (Fig. 6N,N′). Loss of *Pinch* resulted in normal integrin expression in healthy egg chambers (Fig. 6P) and some initial αPS3 enrichment, but no further enrichment as engulfment progressed (Fig. 6Q,Q′). Loss of either *talin* or *Pinch* resulted in engulfment-defective egg chambers (Fig. 6O,R).

Loss of *Syntaxin 5* and *talin* had the strongest and least variable defects in engulfment, so we analyzed their phenotypes further. Antibodies were available against Talin, so we tested the knockdown of *talin* and confirmed that Talin protein was undetectable in *talin^dsRNA^-*expressing egg chambers (Fig. S4G-J). Both *Syntaxin 5* and *talin* knockdowns had strong defects in terms of unengulfed germline and number of particles engulfed (Fig. 6S,T). In particular, the loss of *talin* was extremely strong and resulted in almost no engulfment or apical αPS3 enrichment (Fig. 6T-W). In contrast to the polarity knockdown lines, neither *Syntaxin 5* nor *talin* knockdowns showed mislocalization of αPS3, but they failed to show apical enrichment (Fig. 6U-W). We examined the expression of Draper in *Syntaxin 5^dsRNA^* and *talin^dsRNA^* egg chambers as well, and found no detectable difference in Draper enrichment (data not shown), suggesting that these genes are specific for integrin trafficking. Several other genes we tested had engulfment defects but no effect on integrin enrichment (Table S1), including *Grasp*, *Pkd1* and *Arf6*; most of these are required for integrin endocytosis in other systems, suggesting that endocytosis does not affect integrin enrichment in FCs.

## DISCUSSION

Here, we have investigated integrin function and regulation within engulfing cells. We have shown here that a specific α-subunit is essential for directing the apical localization and function of the integrin heterodimer during engulfment in non-professional phagocytes. We found that αPS3/βPS becomes specifically enriched on the apical surface during engulfment, which suggests that there might be similarities between the epithelial FCs and other polarized epithelial cells that function as phagocytes. αPS3 and βPS are also required for engulfment in the FCs, which suggests that, like in mammals, the same α-subunit functions in engulfment in both professional and non-professional phagocytes and can pair with multiple β-subunits to do so. The loss of cell-polarity genes in the FCs resulted in engulfment defects and integrin-enrichment defects, suggesting that polarization of the cell is required for integrin trafficking in engulfing cells. Cell polarity and integrin trafficking have been linked specifically at the leading edge in migratory cells, and we found here that other genes associated with migration, Golgi orientation, microtubule stabilization and integrin activation are also required for apical αPS3/βPS trafficking and engulfment. To our knowledge, we show the first evidence that *βPS*, *aPKC*, *baz*, *par-6*, *crb*, *Dhc64C*, *Syntaxin 5*, *Clasp* and *Pinch* are required for engulfment.

**Fig. 7.**
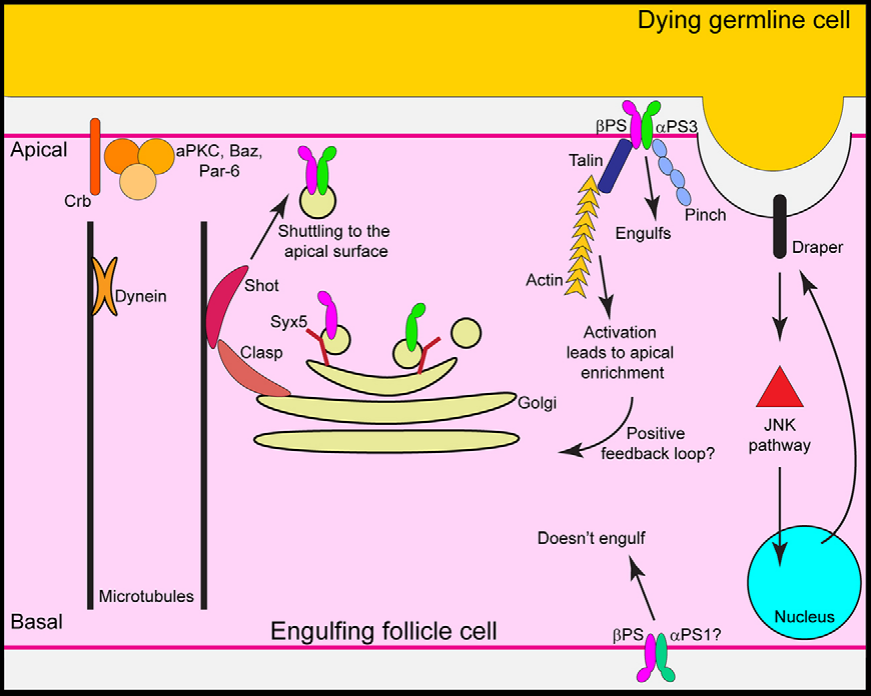
**Model of integrin trafficking in an engulfing cell.** Our results suggest that cell-polarity proteins polarize the microtubules; Shot and Clasp bind to microtubules and shuttle αPS3/βPS to the apical surface. Clasp might also bind to the Golgi and reorient it so that newly synthesized αPS3/βPS is shuttled to the apical surface. Once at the surface, the integrin heterodimer can signal through Talin and Pinch.

We propose the following model (Fig. 7) for integrin trafficking in engulfing cells: cell-polarity proteins polarize the microtubules, and Shot and Clasp bind to microtubules and each other, trafficking αPS3/βPS to the apical surface. Clasp might also bind to the Golgi (Long et al., 2013) and reorient it so that newly synthesized αPS3/βPS is shuttled to the apical surface. Once at the surface, the integrin heterodimer signals through Talin and Pinch, which are required to promote increased αPS3/βPS on the apical surface. Talin might also be required for integrin release from the Golgi, as has been shown in other systems (Martel et al., 2000). Both Talin and Pinch might be required within the cell for integrin trafficking to the apical surface, for downstream integrin signaling and/or for stabilization at the apical surface.

We found it striking that the same α-subunit is required for both migration and engulfment: αPS3 in *Drosophila* and αν in mammals (Dinkins et al., 2008; Shiratsuchi et al., 2012; Nonaka et al., 2013; Law et al., 2015; Tsirmoula et al., 2015). So how are migration and engulfment tied together? Are the mechanisms shared in all migrating and engulfing cells, or only those cells that are already equipped to handle both processes? Epithelial cells are capable of either migration or engulfment when appropriate. Integrins are known for their role in adherence, migration and cancer cell invasion, and regulation of their trafficking and function might play a crucial role in regulating cellular function. It is already known that turnover and regulation of integrin heterodimers determines the speed and direction of a migrating cell, and that, if they are not properly regulated, integrins can contribute to cancer cell invasion (Morse et al., 2014). Migration genes are required for engulfment in professional phagocytes that migrate to the site of the dead cell but, given that FCs do not need to migrate because they are already adjacent to the dying germline, our results suggest that the migration machinery might be integral to the engulfment process itself.

Several types of epithelial cells are crucial for engulfment on a daily basis in organs such as the retina and bronchial tubes. Without proper clearance in these and other organs, debilitating conditions can develop. However, not much is known about how epithelial cells undergo the molecular changes necessary for engulfment. Thus far, we have identified two engulfment receptors, Draper and αPS3/βPS, and the downstream signaling components of the JNK pathway, as being required for engulfment by the FCs. In addition, we have shown that integrins become enriched specifically on the apical surface of the FCs, and we identified several genes required for integrin trafficking, suggesting that the ovary provides a valuable model for studying integrin trafficking and function within a polarized epithelium. Our findings show that the *Drosophila* ovary serves as an excellent model for studying the pathways governing engulfment within epithelial cells.

## MATERIALS AND METHODS

### Fly strains and manipulations

All strains were reared on standard cornmeal molasses fly food at 25°C unless otherwise indicated. For starvation experiments, adult flies were conditioned in new vials containing fly food supplemented with freshly made yeast paste for 1.5-2 days and transferred to apple juice agar vials overnight (Sarkissian et al., 2014). When flies are deprived of nutrients overnight (approximately 16-20 h), egg chambers in all five phases of death can be found (Etchegaray et al., 2012). All strains were obtained from Harvard TRiP (Ni et al., 2008), the Bloomington Stock Center or Vienna *Drosophila* Resource Center unless otherwise indicated (Table S1). *G89* (G00089), a GFP gene trap from FlyTrap (Morin et al., 2001; Etchegaray et al., 2012) was recombined with the FC-specific driver *GR1-GAL4* (Trudi Schüpbach, Princeton University, NJ, USA; Goentoro et al., 2006; Etchegaray et al., 2012) and was crossed to all *UAS* lines. Some dsRNA lines were lethal, so *GR1-GAL4* was combined with *tubulin-GAL80^ts^* and flies were reared at 18°C. Progeny with *tubulin-GAL80^ts^* were transferred to 29°C for 2 days to inactivate GAL80. All dsRNA crosses shown were transferred to 29°C while conditioning and starving. All other lines were reared, conditioned and starved at 25°C. The *puc-lacZ* line was *puc^A251.1^,ry/TM3* (Martín-Blanco et al., 1998; Etchegaray et al., 2012). *drpr^Δ5^* (*draper^−/−^*) (Freeman et al., 2003; Etchegaray et al., 2012) was provided by Estee Kurant (Technion-Israel Institute of Technology, Haifa, Israel) and the *UAS-βPS* lines (Schöck and Perrimon, 2003) were provided by Frieder Schöck (McGill University, Quebec, Canada).

### Antibody staining and microscopy

Flies were dissected in Grace's media and ovaries were fixed and stained as described previously (Tanner et al., 2011). Samples were mounted in VectaShield with DAPI (Vector Labs). Primary antibodies used were: anti-cleaved-Dcp-1 (1:100, Cell Signaling Technology), anti-Dlg [1:100, Developmental Studies Hybridoma Bank (DSHB)], anti-αPS3 (1:300, Shigeo Hayashi, or 1:1000), anti-βPS (1:10, DSHB), anti-Drpr (1:50, DSHB 5D14), anti-β-Gal (1:400, Promega), anti-aPKC (1:1000, Santa Cruz Biotechnology, Inc.), anti-Dynein (1:3, DSHB 2C11), anti-Kinesin (1:100, Cytoskeleton, Inc.), anti-Crumbs (1:25, DSHB Cq4, protocol from Tanentzapf et al., 2000) and anti-Talin (mixture of A22A and E16B, 1:50 each, DSHB). The anti-αPS3 antibody was made using the peptide sequence utilized for the original antibody (Wada et al., 2007) and generated by YenZym (San Francisco, CA). The serum was affinity-purified twice before use. It shows the same expression pattern as the original antibody from the Hayashi lab. Secondary antibodies used were goat-anti-rabbit Cy3, goat-anti-mouse Cy3, goat-anti-mouse Alexa Fluor 647 (Jackson ImmunoResearch), each at 1:100, and goat-anti-rabbit Alexa Fluor 488 (Invitrogen) at 1:200. Egg chambers were imaged on an Olympus FV10i confocal microscope, images were processed using ImageJ and Adobe Photoshop, and figures were made using Adobe Illustrator.

### Western blot staining and quantification

To prepare tissue for the western blot, ten ovaries were homogenized in 150 µl dH_2_O using a motorized pestle before boiling. 10 µl of each sample was loaded into a precast 10% polyacrylamide gel to separate proteins. Separated protein was then transferred onto nitrocellulose membranes. Membranes were immunoprobed with αPS3 (1:2000) and lamin 84.12 (1:10,000), followed by LiCor fluorophore-conjugated secondaries (1:20,000). Blots were imaged and quantified using Image Studio.

### Engulfment quantification

We quantified the percentage of unengulfed germline as previously described (Etchegaray et al., 2012). At least three egg chambers were analyzed for each phase and genotype. To measure uptake directly, we used an antibody raised against cleaved Dcp-1 to mark engulfed particles (Sarkissian et al., 2014), and at least three egg chambers were quantified for each phase (phases 0-4) and genotype. *P*-values were determined using one-way ANOVA and a Bonferroni-Holm post-hoc test.

### Integrin enrichment quantification

To quantify the localization of αPS3 and βPS, lines were drawn from apical to basal through nine cells of each egg chamber and a line plot of the intensity measurements was generated (Fig. S1). The last two microns on either end of the cell were designated as the apical or basal regions. The peak values were identified and the remainder of the cell (cytoplasmic) was averaged; these values were averaged and graphed. These values were normalized to the average intensity from healthy control egg chambers imaged on the same slide at the same settings. At least three egg chambers for each phase and genotype were quantified (phases 1-4). *P*-values were determined using one-way ANOVA and a Bonferroni-Holm post-hoc test.
